# Healthy Eats—Evaluation of a Social Marketing Program Delivered in Primary School Settings in Queensland

**DOI:** 10.3390/ijerph192114415

**Published:** 2022-11-03

**Authors:** Sebastian Isbanner, Julia Carins, Sharyn Rundle-Thiele

**Affiliations:** Social Marketing at Griffith, Griffith Business School, Griffith University, Nathan, QLD 4111, Australia

**Keywords:** nutrition, fruits, vegetables, nutritional knowledge, social marketing, health promotion, schools, children, Queensland

## Abstract

One in four school children in Australia are overweight or obese. In response, the Healthy Eats program was developed, piloted, and delivered using a whole-of-school approach underpinned by the socio-ecological model to increase fruit and vegetable consumption among children aged 8–10 years in regional Queensland, Australia. This research presents an outcome evaluation of the Healthy Eats program using pre–post data collected throughout 2021 (cross-sectional for knowledge and longitudinal for behaviour) from 19 schools to assess whether changes occurred in students’ nutritional knowledge (*n* = 1868 (pre = 933, post = 935)) and fruit and vegetable consumption (*n* = 1042 (pre = 521, post = 521)). Knowledge data was collected via self-reports two weeks prior and immediately after the Nutrition Module. Behavioural data on daily fruit and vegetable consumption was gathered via student passports (i.e., surveys) one week before and for four consecutive weeks after the Nutrition Module. Chi-Square Difference tests and t-Tests were conducted with a significance level set at *p* < 0.05. Across all 19 schools, knowledge of the daily recommended serves of fruit and vegetables improved significantly following participation in the program, aligning knowledge closer to the Australian dietary guidelines. Behavioural results for fruit consumption were favourable, with clear improvements reported. Increases in vegetable consumption were demonstrated in two of the eight schools. A discussion on the knowledge–action gap is provided, including recommendations for future iterations of the Healthy Eats program.

## 1. Introduction

In Australia, about one in four (25%) children and adolescents aged 5–17 are overweight or obese. While overweight and obesity rates in Queensland reflect those of the country [1], obesity rates are increasing at higher rates among Aboriginal and Torres Strait Islander children compared with non-Indigenous children [2,3]. Overall, less than 5% of Queensland children meet the Australian dietary guidelines for recommended serves of fruit and vegetables [4]. The consequences of overweight and obesity can persist into adulthood and increases the risk of cardiovascular disease, Type 2 diabetes, stroke, and poor mental health. Combined with these health risks, there are also growing rates of socio-economic inequality, with overweightness and obesity continuing to increase among Australian children in families with lower socio-economic status [5,6]. Thus, programs that invest into the health and wellbeing of children remain vital to combat a growing problem delivering significant cost to society.

Interventions that incorporate multifactorial approaches are needed to address the complexity of obesity and diet-related health problems [7,8]. Schools represent one key setting for multifactorial public health strategies aiming to lower the prevalence of overweightness and obesity [9,10,11]. School-based programs are effective in influencing children’s learning environments at a young age, thereby facilitating the development of healthy habits, which results in improved health and wellbeing later in life [12,13,14]. Whilst nutrition education alone has been shown to be insufficient to effect change [15], the creation of learning environments that move beyond knowledge to develop skills and stimulate action can be more effective [16]. Previous research shows the greater the number of actions implemented in schools aiming to support and promote healthy eating, the more likely students are to adopt healthy eating practices [17,18]. 

The socio-ecological model accounts for the multifactorial nature of changing eating behaviour in school settings as it recognises the intrapersonal, interpersonal, organisational/settings, community, environment and political influences, and their interactions [19]. Within the socio-ecological model, these levels include the immediate setting (e.g., home, school, workplace) and relationships within and between them; relationships between settings in which the individual person does not participate but which influence the immediate environment (e.g., the education system); generalised patterns that define the substance and structure of other systems (e.g., societies, social groups) but which are modifiable (e.g., by public policy) [19]. The socio-ecological model has been recommended as a comprehensive and holistic approach for the design, implementation, and evaluation of healthy behaviour programs [20,21] as it considers the interaction of behaviours across multiple levels of influence [22,23]. For example, nutrition education is a cornerstone of health behaviour change [24,25,26,27]. Teachers are powerful contributors to students’ learning [28] and are integral to promoting healthy eating habits in a school environment. However, teachers require training, ongoing support and resources to achieve sustained behaviour change program implementation [29]. Parental involvement is crucial as parent behaviour influences what children learn, how children respond to their environment and what children expect of themselves [30,31]. Parental support is important given children consume about 65% of their total energy intake at home [32,33,34]. Furthermore, the content of lunch boxes prepared by parents is directly related to behaviour, performance, achievement, and obesity levels of children [35,36], and studies suggest many parents would prefer their children to eat foods other than what they usually provide to them in a lunchbox at school [37] and that they need support in selecting nutritious, convenient, inexpensive and appealing lunch box food/drinks for children to eat [38]. Creating a positive school environment is vital to support students’ healthy eating habits and attitudes to food. Vegetable gardens have shown to provide an engaging environment that can be used as an instructional tool in a range of subject areas, including nutrition [39]. A range of program and evaluation studies have consolidated evidence for the effectiveness of vegetable school gardens in increasing children’s preference for fruits and vegetables [40,41,42,43,44,45]. As part of the school environment, tuckshops have also been found to influence students’ eating habits [46]. Many foods sold at tuckshops are often characterised by a lower nutritional value (e.g., snack foods). Therefore, it is imperative to encourage tuckshop involvement in promoting healthy eating in schools [47,48]. 

Life Education Queensland (LEQ) developed, piloted, and delivered the Healthy Eats (HE) program using a socio-ecological lens [49]. A formative research phase included a review of literature, surveying parents and carers in North Queensland, observations of the food environment, and stakeholder consultations and co-design sessions. A total of 73 community organisations and members were consulted which comprised (i) various community organisations, neighbourhood centres, community health centres and city councils, industry bodies and community networks, and (ii) community members such as food suppliers, local dieticians, and LEQ educators. 

Following formative research and design, a pilot program was launched. LEQ held consultations with 41 state schools across North Queensland, of which 20 agreed to participate in the pilot in 2019. Schools were identified based on the level of need, and to ensure diversity in the overall sample (i.e., cultural diversity, Aboriginal and Torres Strait Islander Community, small to medium size schools, large schools, outer metropolitan, regional and rural). 

Program components on an individual level included classroom nutrition education (i.e., Nutrition Module to improve knowledge, attitude and beliefs), and fruit and vegetable competition (to foster a positive eating environment and increase fruit and vegetable consumption). On an interpersonal level, families, friends and social networks were addressed via teacher and parental involvement (e.g., Healthy Eats teacher professional development session, regular school newsletter, information sessions and posters). Measures taken on a school and community level included tuckshop menu audits, tuckshop resources, school breakfast program resources, school fruit and vegetable garden assistance, the collaboration of other key community organisations (e.g., Queensland Association of School Tuckshops (QAST)) and the inclusion of Healthy Eats “Brain Breaks” to create consistent daily healthy eating habits. 

The evaluation of the pilot showed promising results, with more than 90% of students correctly identifying the recommended serves of fruit and vegetables, and more than two-thirds able to retain this knowledge ten weeks later. There was a 45% increase in schools with vegetable gardens, and a 50% increase in schools offering breakfast for children. Compliance with the Smart Choices minimum standard increased from 0% to 22% of tuckshops, and a further 17% achieved the highest possible rating [49]. 

Following review and recommendations from pilot schools, the program was revised to include ‘Healthy Eats accreditation’ (to incentivise schools to implement and maintain key healthy eating initiatives), and professional development modules for teaching staff. The revised Healthy Eats program was implemented on a broader scale to schools in Southeast Queensland in 2021. 

The goal of this paper was to present a student-focussed outcome evaluation of HE in schools in North Queensland, focussing on determining whether the main program components achieved their primary goal (i.e., changing students’ knowledge and consumption of the recommended serves of fruit and vegetables). Sharing results from the evaluation is vital to provide insights into program delivery and effectiveness, and unintended consequences, which inform improvement of future programs. 

## 2. Materials and Methods

### 2.1. Study Design

The data used in this evaluation of the Healthy Eats Program were provided to Griffith University by Life Education Queensland (LEQ). The data were collected through self-reports, throughout 2021, which were administered by both teachers and Healthy Eats Educators in schools. Data were collected to assess the learning outcomes of the Nutrition Module (the Knowledge Survey) and to examine the behavioural outcomes of the program (data collected during the Passport Competition). Knowledge Survey data, in the form of knowledge of recommended serves for fruit and vegetables, and knowledge of how to make a healthy snack, were collected cross-sectionally in two stages: two weeks before the start of the module, followed by a post-session survey administered directly after the completion of the module. This research was approved by the researchers’ Human Research Ethics Committee (Ref: 2022/789).

Behavioural data (i.e., Passport Competition) were collected longitudinally in two parts: first, Passport Competition student passports (i.e., surveys) were given to students to record daily (Mon–Fri) serves of fruit and vegetable intake one week prior to the start of the Nutrition Module. After the Nutrition Module, students used Passport Competition student passports for four consecutive weeks to record daily (Mon–Fri) serves of fruit and vegetable intake. 

### 2.2. Population

The schools included in this evaluation were those who elected to participate in the Healthy Eats program in the 2021 program offered by LEQ. Regional schools were invited to participate in the program in recognition of the need for healthy eating programs in these areas. As such, no schools were in big cities, and the schools were public or independent primary schools. The data collected in these pre- and post-surveys asked students to provide demographic information (i.e., age, gender, and ethnicity) and other program-specific background data. Overall, for the Knowledge Survey, 1868 pre-post responses were collected (i.e., pre-program (*n* = 933); post-program (*n* = 935)) from 19 schools (cf. Table 1). Please note that those cases that were removed due to missing values or outliers are still included in this table and will be highlighted in each of the statistical tests following the sample description. 

### 2.3. Data Analysis

Before the start of the statistical analysis, the data were screened for missing values and outliers. To evaluate missing data, pairwise deletion was used to avoid discarding an entire case and therefore, maximise all data that were available on an analysis-by-analysis basis [50,51]. For example, some cases in the individual-level analysis of the Passport Competition may lack pre-post vegetable consumption data but have sufficient data on pre-post fruit consumption. Instead of discarding the entire case, the data on vegetable consumption were retained. 

To correct for outliers, two decision rules were put in place. In Australia, a nationally representative nutrition survey has established the range of intake of fruit and vegetables for children. The mean vegetable intake for 9–11 year old children is 2.3 servings, with a standard deviation (SD) of 2.1 for each individual per day [52]. Based on this national average, we then calculated the maximum possible number of servings a child is likely to eat when it comes to serves of vegetables using the mean value of vegetable servings (i.e., 2.3) plus two standard deviations (following the empirical rule that states 95% of values fall within two standard deviations from the mean). Thus, the estimated maximum number of vegetables (rounded up) is 7 servings per day (2.3 + 2.1 × (2) = 6.5 ≈ 7). If students reported to have eaten equal to or more than 35 serves of vegetables per week (7 × 5 weekdays), the case was discarded as ‘highly unlikely’. Due to the similar pattern of distribution [53] when it comes to the national average of serves of fruit (i.e., 2.2), the same logic was applied (2.2 + 2.1 × (2) = 6.4 ≈ 7). For example, if students reported to have eaten equal to or more than 35 serves of fruits per week (7 × 5 weekdays), the case was discarded as ‘highly unlikely’. It should be noted here that the decision rules established were not intended to be aligned with the recommended daily intake of fruit (two serves) and vegetables (five serves). Rather, they were based on the amount of fruit and vegetable serves children eat on average, which was derived from a large national survey. The decision rules were used as a benchmark to determine what is logically possible for a child to eat daily. This provided a cut-off point to identify outliers in the data set. 

A series of statistical tests were used to analyse the effectiveness of the Healthy Eats program. To provide an overview of the Knowledge Survey sample, basic demographic analyses and means analyses were used, which included Chi-squared difference testing to test whether groups within the pre-post sample were different regarding age, gender, ethnicity, program participation, tuckshop, pathway and vegetable garden. Responses from the Knowledge Survey on knowledge of recommended daily fruit and vegetable servings was analysed using means analyses, Independent Sample t-Tests and One Sample t-Tests. Follow-up tests to assess group differences were conducted, which included one-way analyses of variance (ANOVAs) on gender and pathway (core, ‘halfway’, fully accredited) along with a series of Independent Sample t-Tests on ethnicity (Aboriginal and Torres Strait Islander (ATSI) vs. non-ATSI), program participation (first-timers vs. previously participated) and vegetable garden. 

To test for changes in knowledge of healthy food skills, responses to the open-ended questions asking participants to “Name a healthy snack/how to make a healthy lunchbox snack” were manually coded. The coding scheme was constructed to align with the goals of the program (fruit and vegetable consumption) while minimising the potential for researcher bias. In the first round of coding, ‘Snack name’ was coded as either whole fruits or whole vegetables—or mixed ingredients, in which case the snack was sent to the second round. In the second round, ‘Ingredients to make the snack’ was coded as having fruit ingredients, vegetable ingredients, both fruit and vegetable ingredients, or ‘Other foods’. The category ‘Other’ contains all responses that did not include whole fruits/vegetables. Table 2 below provides some examples of the manual coding that was undertaken. 

To test differences between the groups pre- and post-program, Chi-squared post-hoc tests based on adjusted standardised residuals (i.e., adjusted z-scores) were conducted following Beasley and Schumacker [54]. 

For Passport Competition data, which allowed for matching participants pre-post program, Paired Sample t-Tests in conjunction with One Sample t-Tests were employed on fruit and vegetable consumption. Following data entry and cleaning, descriptive and inferential statistics were estimated. Statistical analysis was conducted using IBM SPSS Statistics (Version 28) for Windows. 

## 3. Results 

### 3.1. Changes in Knowledge of Recommended Daily Serves

**Figure 1:**
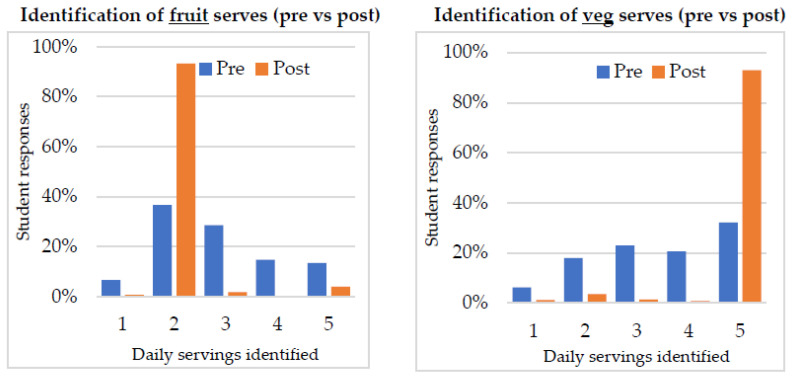
Student responses for recommended number of serves of fruit and veg. per day.

To determine whether participation in HE resulted in changes in knowledge of recommended fruit and vegetable consumption, students’ responses to Questions from the pre-survey with Questions from the post-survey were compared, which asked students to indicate their knowledge on recommended fruit and vegetables serves per day using a five-point Likert-scale. Table 3 and Figure 1 show the students’ responses for the recommended number of serves of fruit and vegetables. 

The results reveal that before the HE program, 36.7% of students correctly identified the recommended number of daily fruit serves. Post-program, the number of students who correctly identified daily fruit serves increased to 93.4%. In a similar vein, the identification of the recommended number of vegetable serves per day increased from 32.1% (pre) to 93.0% (post). An Independent Sample t-Test conducted on combined data from all schools (cf. Table 3) indicated that prior to HE, students reported they should eat 2.92 serves of fruit a day (on average) and post-participation an average of 2.13 serves per day, which was evidently in line with Australian guidelines—a significant change (t(1419) = 18.44, *p* < 0.001). Before the program, students reported they should eat 3.55 serves of vegetables a day (on average). Post-program an average of 4.81 serves per day was evident, which is closer to Australian guidelines—also a significant change (t(1466) = −26.12, *p* < 0.001). This indicates that HE positively influenced students’ knowledge of the recommended serves of fruit and vegetables.

Overall, the results show that all schools but three (School D, H and K) showed a significant change in knowledge of recommended daily fruit serves following participation in the Healthy Eats program. Likewise, all schools but one (School K) observed a significant increase in knowledge of the recommended daily vegetable serves. 

To examine these changes in the context of the key lessons in the program, it was necessary to test if students’ knowledge was in accordance with the recommended daily serves of fruit and vegetables, at either timepoint. Consider two examples: the first being a school where the data show that students’ knowledge of vegetable serves improved and is now close to the recommended number, and the second being a school where the data show that students’ knowledge of vegetable serves also improved but is still some distance from the recommended number of serves. One Sample t-Tests were conducted to determine whether students’ knowledge of the recommended serves was (on average) aligned with dietary guidelines at either point—beforehand, meaning they did not need to change, or afterwards, meaning their knowledge was now accurate.

Table 4 below shows that student knowledge within several schools was already close to the goal of two serves of fruit per day pre-program, including School C (2.5), School H (2.6) and School R (2.6). Overall, in most schools, knowledge regarding the recommended serves of fruit intake moved towards the goal of two serves per day. Particularly strong changes in knowledge towards the goal of two serves of fruit post-program were observed at 12 schools (Schools A, B, E, F, G, I, J, L, M, N, O, P and S). Those schools where knowledge remained different to the mean score of two serves overestimated their number of fruit serves. While still moving towards the goal of two serves of fruit per day, School D displayed the highest post-program mean score of 2.5. Taken together, more than two-thirds (i.e., 68.4%) of schools can be considered as having achieved the goal, as their students can accurately report knowledge of the recommended serves of fruit after participation in the Healthy Eats program. 

When it comes to knowledge of the recommended amount of daily vegetable serves, Table 5 shows that none of the schools were close to the recommendation before program participation. This can be seen by the significant differences between the mean score pre-program compared to the recommended number (or the test value) of 5. Post-program, however, all schools showed positive changes in knowledge and moved towards the goal of five serves per day. Particularly strong changes in knowledge towards the goal of two serves of vegetables post-program were observed at 13 schools (Schools A, B, C, D, E, G, J, M, M, N, O, P, Q and S). The results show that almost half of the schools (i.e., 47.4%) achieved the goal, as their students could accurately report the recommended number of vegetable servings post-program. The other half of schools where student knowledge was still different to the recommended number (or test value) of five servings had all underestimated the number of recommended vegetable servings. While still reporting a positive change of 17.3% towards the goal of five serves of vegetables per day, School K observed the lowest post-program score of 4.3, relative to all other schools. 

### 3.2. Group Differences in Knowledge

To test for group differences in knowledge, a series of one-way analyses of variance (ANOVAs) and Independent Sample t-Tests were conducted (cf. Table 6). Some differences between groups were found prior to Healthy Eats but not after participating in the program. These results suggest that the program may have been making progress, rectifying some of the differences between advantaged and disadvantaged groups. 

### 3.3. Changes in Knowledge of Healthy Food Skills

**Figure 2:**
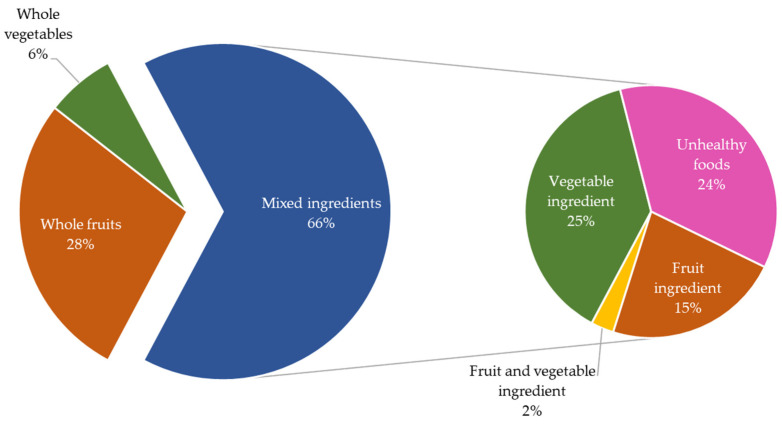
Ingredients within healthy snacks nominated by children (pre-program).

**Figure 3:**
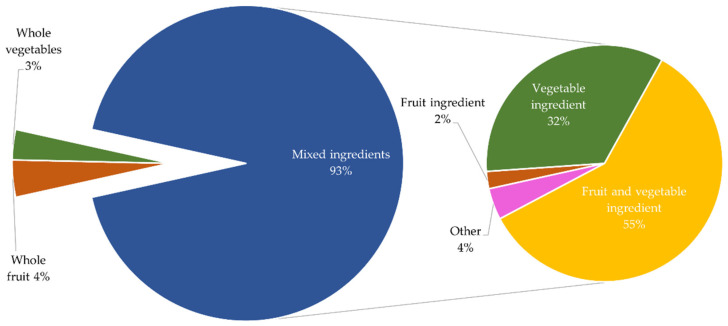
Ingredients within healthy snacks nominated by children (post-program).

A comparison between pre-post data was undertaken for questions asking participants to “Name a healthy snack/how to make a healthy lunchbox snack”. Figure 2 shows that pre-program, responses for all schools combined identified 6% whole vegetables, 28% whole fruits while 66% contained mixed ingredients. In the mixed ingredients category, 25% of all responses included vegetable ingredients, 15% fruit ingredients and 2% both fruit and vegetable ingredients. The remaining 24% contained other (non-fruit and non-vegetable) foods. Figure 3 shows the fruit and vegetable content of the snacks nominated by children.

The results obtained post-program show considerable differences compared to pre-program (see Figure 3 below). 

Firstly, a greater number of healthy snacks was identified, which fall into the ‘Mixed ingredients’ category, representing 93% (vs. 66% pre-program) on average. Of these 93%, 32% included vegetable ingredients, (a 7% points increase compared to pre-program). Importantly, over half of the responses in ‘Mixed ingredients’ category included both fruits and vegetables, (an increase of 53%-points compared to pre-program). Fruit ingredients decreased post-program from 15% to 2%. Lastly, unhealthy foods made up 4% of ‘Other’, which compares favourably to the 24% of unhealthy foods identified pre-session. To test differences between the groups pre- and post-program, Chi-squared post-hoc tests based on adjusted standardised residuals (i.e., adjusted z-scores) were conducted. The post-hoc tests revealed that the healthy lunchbox snacks named by children changed after the program. Firstly, the proportion/percentage of children naming whole fruits and whole vegetables decreased (i.e., whole fruits 28% before vs. 4% after, *p* < 0.001; whole vegetables 6% before vs. 3% after, *p* < 0.001). This was due to substantially more children naming a snack made of several ingredients. Within these, fruit ingredients decreased (15% before vs. 2% after, *p* < 0.001), while vegetable ingredients increased (25% before vs. 32% after, *p* = 0.0019), as did fruit and vegetable ingredients (15% before vs. 55% after, *p* < 0.001). Other (non-fruit or vegetable) ingredients decreased (24% before vs. 4% after, *p* < 0.001).

### 3.4. Changes in Analysis of Passport Competition (Behaviour)

Eight schools reported data for individual students during the Passport Competition, which were included in this analysis. Pre- (*n* = 521) and post-data (*n* = 521) were collected from the same students, therefore allowing pairwise testing. Before commencing the analysis, the data were screened for outliers. Based on the decision rules determined in Section 3, a total of 18 cases (3.5% of the data pool) were identified and removed from the individual level analysis (i.e., 10 × School H, 6 × School S, 1 × School R, 1 × School L). Paired Sample t-Tests indicated that when data was combined for all schools, there were no differences between pre–post reported consumption, both for fruit servings and vegetable servings. Some differences were seen in reported fruit and vegetables consumption between pre- and post-program at some schools (cf. Table 7). 

School G showed a significant decrease in fruit consumption, aligning consumption with recommended daily consumption rates. School L indicated fruit intake increased significantly, exceeding daily recommended consumption rates. In some of the cases, daily fruit consumption remained stable, (Schools F, P, R, S) and was aligned to the recommended levels of two serves per day. 

Two of the eight schools increased vegetable consumption significantly (School F and L). Six schools observed no change in vegetable consumption (Schools G, H, I, P, R and S). 

One-Sample t-Tests were used to examine whether students (on average) reported consumption approaching the recommendations that they have been acquiring knowledge about as part of the Healthy Eats program. It needs to be highlighted that ‘daily’ serves reported only consider children’s consumption at school (and not at home before or after school). Therefore, this is likely to be an underestimation of their daily consumption. This test shows that at most schools, children were meeting their recommended daily fruit intake at school. Even with increases and decreases in consumption (Table 8) consumption became or remained aligned with the daily recommendations. 

For completeness, the one-Sample t-Test was also performed for reported vegetable consumption (see Table 9 below). Again, ‘daily’ serves reported only consider children’s consumption at school and given it is expected that many children would consume vegetables as part of an evening meal at home, this is likely to be a substantial underestimate of their daily consumption. Indeed, this analysis shows that despite large proportional increases in vegetable consumption at school, children were not close to meeting their recommended daily vegetable intake at school (nor is it expected that they would do so). 

The lack of significant change for some of the schools (cf. Table 7) could be the result of combining responses from students who increased their consumption (towards the recommended daily serves), with the responses from students who decreased consumption (towards the recommended daily serves). These two types of changes cancel each other out—creating an average of ‘no change’. To assess how many students reported increases in consumption, decreases in consumption or no change in consumption, Table 10 divides mean consumption into three categories: (1) increase, (2) no change and (3) decrease. 

For fruit, across all schools, more children reported increases in consumption. Increases in consumption were more often seen at School F, School H, School L, School P and School S—where consumption was mostly below two serves prior to the program, and at or above two serves afterwards. Decreases in consumption were more often seen at School I, School R and in particular School G following the program. Fruit consumption at these schools was above two serves prior to the program, and only marginally below two serves afterwards. For vegetables, across all schools, more children reported increases in consumption. Increases in consumption were more often seen at all schools (except School S and School G). Of note are School F, School I and School L, all of which posted strong positive changes in the number of vegetable serves consumed. There are not enough responses in each category (increase, no change, decrease) to perform a robust statistical analysis, but this table suggests that for fruit, changes in both directions resulted in children’s consumption becoming more aligned with the daily recommendations. For vegetable consumption, more children increased their consumption, but a large proportion reported decreases in consumption.

## 4. Discussion

The Healthy Eats Program, developed and delivered by Life Education Queensland (LEQ), aimed to empower students to make healthier food choices by developing and sustaining a whole-of-school approach. This outcome evaluation was undertaken to (1) assess the extent to which students have retained or changed knowledge about daily fruit and vegetable consumption recommendations and (2) explore whether students’ fruit and vegetable consumption improved after the program. Within most schools, knowledge of the daily recommended serves of fruit improved, aligning with Australian recommendations [55]. Knowledge of recommended vegetable serves improved in most schools, but for several schools, knowledge remained some distance from recommended Australian dietary guideline levels [55]. The findings support the positive effect of the Healthy Eats program on students’ healthy eating knowledge. These changes should be recognised as a successful outcome.

The program was more effective at aligning fruit consumption closer to recommended daily consumption levels than it was for vegetable consumption. It should be noted that increasing children’s consumption of vegetables is the most important goal of the program, recognising that less than 6% of Queensland students consume the recommended serves each day. Other studies have noted the difficulties associated with improving vegetable intake compared to fruit [56]. While some positive effects were observed for vegetable consumption and significant enhancement of knowledge relating to vegetable intake occurred, continued effort will be needed to understand how to align vegetable consumption more closely to recommended daily intake rates. 

The positive knowledge changes without subsequent improvement in vegetable consumption resonate with the mixed body of evidence on school-based nutrition programs. For example, similar to the findings of the Healthy Eats program, Long and Stevens [57] found increases in nutritional knowledge in the program group, which did not translate into food choices. On the other hand, a few evaluations of school-based nutrition programs report that increases in students’ nutritional knowledge were reflected in subsequent food choices (e.g., [58,59,60,61]). The mixed evidence suggests that nutrition education is necessary, yet not always sufficient in effecting desired behaviour changes. 

The reasons for the knowledge–action gap may be manifold. In the case of this program, ‘Daily’ serves reported only consider children’s consumption at school (and not at home before or after school). Therefore, this is likely to be an underestimation of their daily consumption. This is a substantial consideration given Australian children aged 9–11 consume a large proportion of their daily vegetable intake (close to 60%) during dinner [62]. For fruit, the opposite has been observed, where the majority is consumed across the day, during lunch, and morning and afternoon snacks (60% of daily intake) [62]. This suggests that programs aiming to change children’s healthy eating may need to capture behaviour across the entire day to determine whether the program has been effective or that more effort needs to be directed at increasing the proportion of people who consume vegetables throughout the day. Further, six weeks is suggested as the ideal time interval to establish behaviour change [63], which suggests that extending the passport competition to 6 weeks warrants trialling. 

Nutrition knowledge (and by extension—nutrition literacy) is a broad concept, covering the knowledge, skills, and competence required to achieve nutritional health [64]. Two components relevant to this study are declarative knowledge (e.g., knowing a person should eat two fruit and five vegetables a day) and procedural knowledge (e.g., knowing how to construct a healthy snack using fruit and vegetables) [65,66]. Declarative knowledge may be a prerequisite for procedural knowledge; however, declarative knowledge does not automatically translate into procedural knowledge, and procedural knowledge does not always translate into improved dietary behaviours [67]. There is concern among community nutritionists and others that many children have poor experiential knowledge of food and have few buying and preparation skills [68]. This emphasises the need for school programs to develop both knowledge and skills. 

Furthermore, parents’ actions are a critical determinant of children’s fruit and vegetable consumption, through both role modelling of consumption [69] and as the adults who control availability and accessibility within the home [70]. The Healthy Eats program provided engagement strategies and resources to parents, and future evaluations provides an avenue to understand whether parental involvement in these program components is strong, and to what degree these components generate support for children’s behavioural change. Children develop within a wider environment, and are influenced by many factors within their community, neighbourhood, or area. These differences due to their background [71] have a bearing on children’s knowledge and behaviour prior to program involvement [72,73]. Future research should include a deeper analysis of the importance of these background factors to determine how they might impact program effectiveness. This underscores the need to take a socio-ecological view to improving children’s healthy eating behaviours to ensure that the school and home environments support children to develop healthy eating behaviours, and do not perpetuate existing inequalities. However, supportive infrastructure is needed. Systems-based policy and actions to improve the school food environment will support individual based strategies such and nutrition education and skills-based learning [74].

Finally, longer exposure to program strategies are likely to be needed to create stronger effects on behaviour change [63], as well as repeat exposure over time to retain those behaviours through adolescence into adulthood. This means that programs need to be planned to cover the educational lifecycle of children, from the first 2 years of life into school-aged children [75,76,77,78], and evaluation would need to include replication with longitudinal design [79,80,81]. A longer timeframe will also allow for environmental strategies to contribute to behaviour change. Other studies that have adopted a whole of school approach have demonstrated positive effects over the length of the school year [82].

Outcomes observed in the 2021 Healthy Eats program evaluation deliver support for the viability of the socio-ecological model as a framework that can be applied within social marketing programs that aim to promote healthy eating in school environments. In summary, the evaluation of the Healthy Eats program indicates there were increases in students’ knowledge of fruit and vegetable recommendations, which only partially translated into behavioural shifts. Opportunities to enhance program effects are evident from the outcome evaluation, which would be a fruitful area for further work. For example, Social Cognitive Theory (SCT) [83] has shown promise in multiple school-based nutrition programs [84,85]. Future research should address how the socio-ecological model may be complemented by SCT to enhance program effects. 

The findings of this evaluation should be considered in light of any limitations. In the Knowledge Survey, some questions were not always worded the same in both the pre- and post-surveys. This makes it difficult to make direct comparisons between the time points. Future data collections should consider how questions can be worded in age-appropriate ways, but not so that they increase the risk of social desirability bias or curtail the richness of the data and it should ensure that repeated measure designs are used. 

For the behavioural data (the passport data), an important limitation was the issue of outliers detected in the data set, which had the potential to distort trends in the data. To minimise the issue of outliers, statistical detection techniques were used to identify and discard serious outliers. Second, the issue of missing data, particularly in the analysis of the Passport Competition, meant that pre-post comparisons were not feasible for all schools. 

The current evaluation considered whether Healthy Eats program components (e.g., modules delivered in primary school settings) achieved intended changes in knowledge of recommended fruit and vegetable consumption and the degree that influencing student knowledge supported behavioural change. Future research is needed to evaluate and appreciate the outcomes and lasting impact derived from other components of the program (e.g., vegetable gardens, parent initiatives and policy changes). For example, a better understanding of how tuck-shop menu audits and vegetable gardens each contribute to initial and lasting behaviour change and behaviour maintenance consistent with recommended daily fruit and vegetable consumption guidelines are also needed to further enhance program improvement and future program implementation.

## 5. Conclusions

The outcome evaluation of Healthy Eats Program, delivered by Life Education Queensland (LEQ), showed improved knowledge of the recommended daily number of fruit and vegetable serves following the program, and some improvements to fruit and vegetable consumption behaviour. Overall, this evaluation supports the effectiveness and importance of conducting classroom and school-based initiatives to increase healthy eating knowledge. 

## Figures and Tables

**Table 1:** Sample demographics–Nutrition Module (Knowledge Survey).

Characteristic	Category	Pre		Post		χ^2^
		*n*		*n*		
Gender	Male	422	44.8%	437	46.5%	χ^2^ (2) = 1.037; *p* = 0.595
	Female	467	49.5%	448	47.7%	
	Prefer not to say	44	4.7%	50	5.3%	
	Missing	10	1.1%	4	0.4%	
	Total	943	100.0%	939	100.0%	
Age	8 years-old	3	0.3%	3	0.3%	χ^2^ (5) = 0.031; *p* = 1.000
	9 years-old	118	12.5%	116	12.4%	
	10 years-old	599	63.5%	598	63.7%	
	11 years-old	196	20.8%	194	20.7%	
	12 years-old	23	2.4%	22	2.3%	
	13 years-old	1	0.1%	1	0.1%	
	Missing	3	0.3%	5	0.5%	
	Total	943	100.0%	939	100.0%	
Ethnicity	Non-indigenous	732	77.6%	714	76.0%	χ^2^ (3) = 32.039; *p* < 0.001 *
	Aboriginal	65	6.9%	118	12.6%	
	Torres Strait Islander	84	8.9%	39	4.2%	
	both Aboriginal and Torres Strait Islander	56	5.9%	58	6.2%	
	Missing	6	0.6%	10	1.1%	
	Total	943	100.0%	939	100.0%	
Program participation	First timer	386	40.9%	387	41.2%	χ^2^ (1) = 0.015; *p* = 0.901
	Previously attended	557	59.1%	552	58.8%	
	Missing	0	0%	0	0%	
	Total	943	100%	939	100%	
Pathway	Core	405	42.9%	399	42.5%	χ^2^ (2) = 0.043; *p* = 0.979
	Halfway/in progress	288	30.5%	290	30.9%	
	Full/completed	250	26.5%	250	26.6%	
	Missing	0	0%	0	0%	
	Total	943	100%	939	100%	
Tuckshop	No	77	8.2	78	8.3%	χ^2^ (1) = 0.012; *p* = 0.911
	Yes	866	91.8	861	91.7%	
	Missing	0	0%	0	0%	
	Total	943	100%	939	100%	
Vegetable garden	No	248	26.3%	249	26.5%	χ^2^ (1) = 0.012; *p* = 0.914
	Yes	695	73.7%	690	73.5%	
	Missing	0	0%	0	0%	
	Total	943	100%	939	100%	

* Post-program, almost twice as many Aboriginal students and Torres Strait Islander students participated in the survey compared to the pre-session survey.

**Table 2:** Coding of ingredients listed in children’s’ descriptions of healthy snacks.

Coding	Label	Response Examples
Round 1	(1) Whole fruits	Apple, banana, orange, strawberries, peach, plums, grapes
	(2) Whole vegetables	Carrot, cucumber, celery, corn, tomato, capsicum
	(3) Mixed Ingredients	[Coded in Round 2]
Round 2	(3) Fruit ingredient	“Banana, milk, flour, eggs” “Apple, peanut butter”
	(4) Vegetable ingredient	“Carrot, peanut butter”; “Chicken, lettuce, cheese, bread”
	(5) Fruit and vegetable ingredient	“Celery, cream, sultanas”; “Celery, peanut butter, sultanas”
	(6) Other foods	Crackers and cheese, meat, jam and bread, pastry, ham

**Table 3:** Independent Samples t-Test results—Nutrition Module.

		Pre-Program		Post-Program				
		*n*	Mean	*n*	Mean	t(df)	*p*	Diff
School A	Fruit serves	42	3.0	41	2.2	4.235 (66)	<0.001	⏺ ↓
	Veg serves	41	3.6	41	5.0	−6.418 (45)	<0.001	⏺ ↑
School B	Fruit serves	87	2.9	84	2.0	7.796 (86)	<0.001	⏺ ↓
	Veg serves	84	3.8	85	5.0	−10.129 (85)	<0.001	⏺ ↑
School C	Fruit serves	56	2.5	55	2.1	2.221 (83)	0.029	⏺ ↓
	Veg serves	56	3.4	55	4.7	−5.949 (100)	<0.001	⏺ ↑
School D	Fruit serves	11	2.8	12	2.5	0.611 (21)	0.547	-
	Veg serves	12	2.8	12	4.8	−4.642 (22)	<0.001	⏺ ↑
School E	Fruit serves	19	2.7	20	2.2	1.648 (26)	0.102	-
	Veg serves	17	2.9	20	4.9	−5.171 (22)	<0.001	⏺ ↑
School F	Fruit serves	13	2.9	13	2.0	2.382 (12)	0.026	⏺ ↓
	Veg serves	12	4.0	13	5.0	−2.449 (11)	0.018	⏺ ↑
School G	Fruit serves	66	2.9	67	2.0	5.398 (78)	<0.001	⏺ ↓
	Veg serves	64	3.2	66	5.0	−10.04 (72)	<0.001	⏺ ↑
School H	Fruit serves	38	2.6	40	2.2	1.95 (70)	0.053	-
	Veg serves	40	4.3	40	4.8	−2.38 (74)	0.020	⏺ ↑
School I	Fruit serves	18	3.1	18	2.2	3.083 (31)	0.004	⏺ ↓
	Veg serves	18	3.9	18	5.0	−4.486 (17)	<0.001	⏺ ↑
School J	Fruit serves	132	3.2	131	2.2	8.04 (206)	<0.001	⏺ ↓
	Veg serves	130	3.4	132	4.7	−9.927 (214)	<0.001	⏺ ↑
School K	Fruit serves	22	2.8	22	2.3	1.519 (42)	0.136	-
	Veg serves	22	3.6	22	4.3	−1.618 (42)	0.113	-
School L	Fruit serves	15	3.4	15	2.0	5.957 (14)	<0.001	⏺ ↓
	Veg serves	15	3.4	15	4.8	−4.537 (28)	<0.001	⏺ ↑
School M	Fruit serves	40	2.8	40	2.1	3.603 (54)	0.001	⏺ ↓
	Veg serves	40	3.5	40	4.8	−5.638 (70)	<0.001	⏺ ↑
School N	Fruit serves	20	2.9	19	2.2	2.674 (34)	0.012	⏺ ↓
	Veg serves	20	3.5	19	4.8	−4.591 (32)	<0.001	⏺ ↑
School O	Fruit serves	63	3.3	63	2.1	6.615 (85)	<0.001	⏺ ↓
	Veg serves	63	3.7	64	4.8	−6.013 (93)	<0.001	⏺ ↑
School P	Fruit serves	69	2.8	63	2.0	6.441 (75)	<0.001	⏺ ↓
	Veg serves	68	3.2	63	4.8	−9.492 (106)	<0.001	⏺ ↑
School Q	Fruit serves	78	2.7	78	2.3	2.822 (139)	0.005	⏺ ↓
	Veg serves	78	3.2	79	4.7	−8.163 (127)	<0.001	⏺ ↑
School R	Fruit serves	56	2.6	57	2.2	2.352 (92)	0.020	⏺ ↓
	Veg serves	57	4.4	57	4.7	−2.098 (106)	0.038	⏺ ↑
School S	Fruit serves	85	3.2	87	2.1	9.071 (132)	<0.001	⏺ ↓
	Veg serves	84	3.5	88	4.8	−8.666 (147)	<0.001	⏺ ↑
Total	Fruit serves	930	2.9	925	2.1	18.44 (1419)	<0.001	⏺ ↓
	Veg serves	921	3.5	929	4.8	26.12 (1466)	<0.001	⏺ ↑

*Note*: Significant differences are highlighted with a black dot (i.e., ⏺), followed by an up- or down-arrow (e.g., ↑) to signify the direction (i.e., increase or decrease).

**Table 4:** One Sample t-Test results fruit—Nutrition Module.

Fruit Serves (Test Value = 2)								
School		*n*	Mean	t(df)	*p*	Diff	Mean Diff	Δ
School A	pre	42	3.0	5.755 (41)	<0.001	⏺	1.0	−28.3%
	post	41	2.2	1.432 (40)	0.08	-	0.1	
School B	pre	87	2.9	7.796 (86)	<0.001	⏺	0.9	−29.8%
	post	84	2.0	n/a *	-	-	-	
School C	pre	56	2.5	3.365 (55)	<0.001	⏺	0.5	−14.8%
	post	55	2.1	1.63 (54)	0.054	-	0.1	
School D	pre	11	2.8	2.043 (10)	0.034	⏺	0.8	−11.3%
	post	12	2.5	1.483 (11)	0.083	-	0.5	
School E	pre	19	2.7	2.281 (18)	0.017	⏺	0.7	−27.0%
	post	19	2.0	n/a *	-	-	-	
School F	pre	13	2.9	2.382 (12)	0.017	⏺	0.8	−29.8%
	post	13	2.0	n/a *	-	-	-	
School G	pre	66	2.9	5.976 (65)	<0.001	⏺	0.8	−28.4%
	post	67	2.0	1.000 (66)	0.16	-	0.0	
School H	pre	38	2.6	3.822 (37)	<0.001	⏺	0.6	−13.7%
	post	40	2.2	1.842 (39)	0.037	⏺	0.2	
School I	pre	18	3.1	4.486 (17)	<0.001	⏺	1.1	−29.1%
	post	18	2.2	1.000 (17)	0.166	-	0.2	
School J	pre	132	3.2	10.89 (131)	<0.001	⏺	1.2	−31.7%
	post	131	2.2	2.998 (130)	0.002	⏺	0.2	
School K	pre	22	2.8	3.645 (21)	<0.001	⏺	0.8	−17.7%
	post	22	2.3	1.322 (21)	0.100	-	0.3	
School L	pre	15	3.4	5.957 (14)	<0.001	⏺	1.4	−41.2%
	post	15	2.0	n/a *	-	-	-	
School M	pre	40	2.8	4.365 (39)	<0.001	⏺	0.8	−25.7%
	post	40	2.1	0.902 (39)	0.186	-	0.1	
School N	pre	20	2.9	3.943 (19)	<0.001	⏺	0.9	−25.5%
	post	19	2.2	1.000 (18)	0.165	-	0.2	
School O	pre	63	3.3	8.001 (62)	<0.001	⏺	1.3	−34.0%
	post	63	2.2	2.097 (62)	0.02	⏺	0.2	
School P	pre	70	2.8	6.445 (69)	<0.001	⏺	0.8	−28.7%
	post	70	2.0	−0.575 (69)	0.284	-	0.0	
School Q	pre	78	2.7	5.574 (77)	<0.001	⏺	0.7	−16.4%
	post	77	2.3	2.982 (76)	0.002	⏺	0.3	
School R	pre	56	2.6	4.172 (55)	<0.001	⏺	0.6	−16.0%
	post	57	2.2	2.269 (56)	0.014	⏺	0.2	
School S	pre	85	3.2	11.437 (84)	<0.001	⏺	1.2	−34.9%
	post	87	2.1	1.919 (86)	0.029	⏺	0.1	
Total	pre	930	2.9	24.479 (929)	<0.001	⏺	0.9	−27.6%
	post	925	2.1	6.688 (924)	<0.001	⏺	0.1	

*Note:* Significant differences are highlighted with a black dot (i.e., ⏺). * The t value was not computed due to a standard deviation of zero (all responses were identical).

**Table 5:** One Sample t-Test results vegetables—Nutrition Module.

Vegetable Serves (Test Value = 5)								
School		*n*	Mean	t(df)	*p*	Diff	Mean Diff	Δ
School A	pre	41	3.6	−6.84 (40)	<0.001	⏺	−1.4	37.1%
	post	41	5.0	−1.000 (40)	0.162	-	0.0	
School B	pre	84	3.8	−10.288 (83)	<0.001	⏺	−1.2	30.3%
	post	85	5.0	−1.000 (84)	0.16	-	0.0	
School C	pre	56	3.4	−9.023 (55)	<0.001	⏺	−1.6	38.1%
	post	55	4.7	−2.257 (54)	0.014	⏺	−0.3	
School D	pre	12	2.8	−6.413 (11)	<0.001	⏺	−2.3	72.7%
	post	12	4.8	−1.000 (11)	0.169	-	−0.3	
School E	pre	17	2.9	−6.104 (16)	<0.001	⏺	−2.1	70.1%
	post	19	5.0	n/a *	-	-	-	
School F	pre	12	4.0	−2.449 (11)	0.016	⏺	−1.0	25.0%
	post	13	5.0	n/a *	-	-	-	
School G	pre	64	3.2	−10.669 (63)	<0.001	⏺	−1.8	54.7%
	post	66	5.0	−1.000 (65)	0.161	-	0.0	
School H	pre	40	4.3	−4.521 (39)	<0.001	⏺	−0.7	10.4%
	post	40	4.8	−1.94 (39)	0.03	⏺	−0.2	
School I	pre	18	3.9	−4.486 (17)	<0.001	⏺	−1.1	26.9%
	post	18	5.0	n/a *	-	-	-	
School J	pre	130	3.4	−13.811 (129)	<0.001	⏺	−1.6	41.1%
	post	132	4.7	−3.546 (131)	<0.001	⏺	−0.3	
School K	pre	22	3.6	−5.257 (21)	<0.001	⏺	−1.4	17.3%
	post	22	4.3	−2.46 (21)	0.011	⏺	−0.7	
School L	pre	15	3.4	−6.808 (14)	<0.001	⏺	−1.6	41.2%
	post	15	4.8	−1.000 (14)	0.167	-	−0.2	
School M	pre	40	3.5	−8.051 (39)	<0.001	⏺	−1.5	37.4%
	post	40	4.8	−1.711 (39)	0.048	⏺	−0.2	
School N	pre	20	3.5	−6.097 (19)	<0.001	⏺	−1.5	38.3%
	post	19	4.8	−1.000 (18)	0.165	-	−0.2	
School O	pre	63	3.7	−7.792 (62)	<0.001	⏺	−1.3	31.5%
	post	64	4.8	−1.93 (63)	0.029	⏺	−0.2	
School P	pre	69	3.3	−11.847 (68)	<0.001	⏺	−1.8	49.5%
	post	70	4.9	−1.857 (69)	0.034	⏺	−0.1	
School Q	pre	78	3.2	−11.303 (77)	<.001	⏺	−1.8	46.6%
	post	78	4.7	−2.926 (77)	0.002	⏺	−0.3	
School R	pre	57	4.4	−4.481 (56)	<0.001	⏺	−0.6	9.0%
	post	57	4.7	−2.32 (56)	0.012	⏺	−0.3	
School S	pre	84	3.5	−12.053 (83)	<0.001	⏺	−1.5	35.9%
	post	88	4.8	−2.35 (87)	0.011	⏺	−0.2	
Total	pre	921	3.6	36.791 (920)	<0.001	⏺	1.6	33.0%
	post	929	4.8	116.744 (928)	<0.001	⏺	2.8	

*Note:* Significant differences are highlighted with a black dot (i.e., ⏺). * The t value was not computed due to a standard deviation of zero (all responses were identical).

**Table 6:** Group differences in knowledge.

	Group Differences						
Group			*n*	Mean	F/t(df)	*p*	Diff
Gender	Fruit	Males (pre) ^a^	418	3.02	F(2, 917) = 3.07	*p* < 0.05	⏺
		Female (pre) ^b^	461	2.83			
		Prefer not to say (pre) ^ab^	41	2.90			
		Males (post) ^a^	430	2.17	F(2, 918) = 1.29	*p* = 0.28	
		Female (post) ^a^	444	2.10			
		Prefer not to say (post) ^a^	47	2.15			
	Vegetable	Males (pre) ^a^	409	3.50	F(2, 908) = 0.42	*p* = 0.66	
		Female (pre) ^a^	459	3.57			
		Prefer not to say (pre) ^a^	43	3.60			
		Males (pre) ^a^	431	4.77	F(2, 922) = 2.63	*p* = 0.07	
		Female (pre) ^a^	444	4.86			
		Prefer not to say (pre) ^a^	50	4.68			
Ethnicity	Fruit	Non-ATSI (pre) ^a^	725	2.86	t(293.7) = −2.72	*p* < 0.05	⏺
		ATSI (pre) ^b^	205	3.13			
		Non-ATSI (post) ^a^	710	2.11	t(299.9) = −1.72	*p* = 0.09	
		ATSI (post) ^a^	215	2.20			
	Vegetable	Non-ATSI (pre) ^a^	717	3.55	t(919) = 0.07	*p* = 0.94	
		ATSI (pre) ^a^	204	3.54			
		Non-ATSI (post) ^a^	708	4.82	t(927) = 0.72	*p* = 0.47	
		ATSI (post) ^a^	221	4.78			
First-timers vs. previously participated	Fruit	First-timers (pre) ^a^	381	2.99	t(928) = 1.64	*p* = 0.10	
		Prev. participated (pre) ^a^	549	2.87			
		First-timers (post) ^a^	384	2.15	t(923) = 0.71	*p* = 0.48	
		Prev. participated (post) ^a^	541	2.12			
	Vegetable	First-timers (pre) ^a^	379	3.42	t(919) = −2.40	*p* < 0.05	⏺
		Prev. participated (pre) ^b^	542	3.63			
		First-timers (post) ^a^	386	4.82	t(927) = 0.50	*p* = 0.61	
		Prev. participated (pre) ^a^	543	4.80			
Pathway	Fruit	Core (pre) ^a^	401	3.01	F(2, 927) = 2.15	*p* = 0.12	
		In progress (pre) ^a^	282	2.85			
		Full accreditation (pre) ^a^	247	2.85			
		Core (post) ^a^	393	2.08	F(2, 922) = 3.10	*p* = 0.05	
		In progress (post) ^a^	283	2.17			
		Full accreditation (post) ^a^	249	2.18			
	Vegetable	Core (pre) ^ab^	396	3.56	F(2, 918) = 5.80	*p* < 0.05	⏺
		In progress (pre) ^a^	283	3.71			
		Full accreditation (pre) ^b^	242	3.33			
		Core (post) ^a^	396	4.82	F(2, 926) = 2.15	*p* = 0.12	
		In progress (post) ^a^	284	4.74			
		Full accreditation (post) ^a^	249	4.87			
Vegetable garden	Fruit	No vegetable garden (pre) ^a^	244	2.98	t(928) = 1.03	*p* = 0.30	
		Vegetable garden (pre) ^a^	686	2.90			
		No vegetable garden (post) ^a^	247	2.14	t(923) = 0.23	*p* = 0.82	
		Vegetable garden (post) ^a^	678	2.13			
	Vegetable	No vegetable garden (pre) ^a^	241	3.34	t(919) = −2.98	*p* < 0.05	⏺
		Vegetable garden (pre) ^b^	680	3.62			
		No vegetable garden (post) ^a^	248	4.82	t(927) = 0.23	*p* = 0.82	
		Vegetable garden (post) ^a^	681	4.81			

^a, b^ Groups with the same superscript letter are not statistically different from each other. *Note:* Significant differences are highlighted with a black dot (i.e., ⏺).

**Table 7:** Individual level Paired Samples t-Test results—Passport Competition.

		Pre		Post				
School		*n*	Mean (Daily)	*n*	Mean (Daily)	t(df)	*p*	Diff
School F	Fruit serves	14	1.8	14	2.5	−1.912 (13)	0.078	-
	Veg serves	14	1.2	14	3.8	−8.597 (13)	<0.001	⏺ ↑
School G ^1^	Fruit serves	29	2.7	29	2.0	3.335 (28)	0.002	⏺ ↓
	Veg serves	29	2.4	29	1.6	1.984 (28)	0.057	-
School H	Fruit serves	37	3.0	37	3.1	−0.374 (36)	0.711	-
	Veg serves	37	3.0	37	3.1	−0.603 (36)	0.551	-
School I	Fruit serves	18	1.7	18	1.5	0.573 (17)	0.574	-
	Veg serves	18	0.3	18	0.6	−1.534 (17)	0.143	-
School L	Fruit serves	15	2.1	15	2.9	−2.663 (14)	0.019	⏺ ↑
	Veg serves	15	1.6	15	2.4	−2.755 (14)	0.015	⏺ ↑
School P	Fruit serves	18	1.7	18	1.8	−0.296 (17)	0.771	-
	Veg serves	18	1.0	18	1.6	−1.925 (17)	0.071	-
School R	Fruit serves	67	2.1	67	2.1	−0.161 (66)	0.873	-
	Veg serves	68	2.5	68	2.6	−0.85 (67)	0.398	-
School S	Fruit serves	63	2.0	63	2.1	−0.686 (62)	0.495	-
	Veg serves	61	1.9	.61	1.8	0.67 (60)	0.505	-
Total	Fruit serves	261	2.2	261	2.2	−0.606 (260)	0.545	-
	Veg serves	260	2.0	260	2.2	−1.71 (259)	0.089	-

^1^ School G pre-survey was based on the whole day, not just at school so their pre- and post-scores may not be directly comparable. *Note:* Significant differences are highlighted with a black dot (i.e., ⏺), followed by an up- or down-arrow (e.g., ↑) to signify the direction (i.e., increase or decrease).

**Table 8:** Increases, decreases and no change in fruit and vegetable consumption at each school.

Fruit Serves (Reported Consumption Compared to 2 Serves *)								
School		*n*	Mean	Daily Mean	t(df)	*p*	Diff	Δ
School F	Pre	14	9.1	1.8	−0.637 (13)	0.535	-	35.2%
	Post	14	12.4	2.5	2.148 (13)	0.051	-	
School G ^1^	Pre	29	13.5	2.7	3.313 (28)	0.003	⏺	−26.8%
	Post	29	9.9	2.0	−0.146 (28)	0.885	-	
School H	Pre	38	14.7	3.0	4.637 (37)	<0.001	⏺	5.0%
	Post	37	15.5	3.1	5.924 (36)	<0.001	⏺	
School I	Pre	18	8.3	1.7	−1.696 (17)	0.108	-	−7.4%
	Post	18	7.7	1.5	−3.636 (17)	0.002	⏺	
School L	Pre	15	10.5	2.1	0.356 (14)	0.727	-	39.9%
	Post	15	14.7	2.9	3.443 (14)	0.004	⏺	
School P	Pre	18	8.3	1.7	−1.182 (17)	0.253	-	6.0%
	Post	18	8.8	1.8	−1.582 (17)	0.132	-	
School R	Pre	67	10.5	2.1	0.838 (66)	0.405	-	3.2%
	Post	68	10.8	2.1	1.365 (67)	0.177	-	
School S	Pre	63	10.1	2.0	0.079 (62)	0.937	-	8.2%
	Post	64	10.9	2.1	1.09 (63)	0.280	-	
Total	Pre	261	11.01	2.2	2.770 (260)	0.006	⏺	3.0%
	Post	263	11.34	2.3	3.932 (262)	<0.001	⏺	

^1^ School G pre-survey was based on the whole day, not just at school so their pre- and post-scores may not be directly comparable. * Based on goal of 2 serves of fruits per day (×5 weekdays). *Note:* Significant differences are highlighted with a black dot (i.e., ⏺).

**Table 9:** Increases, decreases and no change in fruit and vegetable consumption at each school.

Vegetable Serves (Reported Consumption Compared to 5 Serves *)								
School		*n*	Mean	Daily Mean	t(df)	*p*	Diff	Δ
School F	Pre	14	6.2	1.2	−17.919 (13)	<0.001	⏺	205.2%
	Post	14	18.9	3.8	−8.749 (13)	<0.001	⏺	
School G	Pre	29	12.1	2.4	−7.661 (28)	<0.001	⏺	−33.6%
	Post	29	8.0	1.6	−13.153 (28)	<0.001	⏺	
School H	Pre	38	15.1	3.0	−9.779 (37)	<0.001	⏺	4.4%
	Post	37	15.7	3.1	−7.663 (36)	<0.001	⏺	
School I	Pre	18	1.4	0.3	−42.996 (17)	<0.001	⏺	107.9%
	Post	18	2.9	0.6	−29.341 (17)	<0.001	⏺	
School L	Pre	15	8.2	1.6	−9.577 (14)	<0.001	⏺	48.5%
	Post	15	12.1	2.4	−7.349 (14)	<0.001	⏺	
School P	Pre	18	5.1	1.0	−14.352 (17)	<0.001	⏺	54.7%
	Post	18	7.8	1.6	−14.507 (17)	<0.001	⏺	
School R	Pre	68	12.3	2.5	−16.614 (67)	<0.001	⏺	5.0%
	Post	68	12.9	2.6	−15.474 (67)	<0.001	⏺	
School S	Pre	61	9.5	1.9	−13.122 (60)	<0.001	⏺	−0.7%
	Post	64	9.4	1.8	−14.54 (63)	<0.001	⏺	
Total	Pre	251	10.64	2.1	−29.102 (250)	<0.001	⏺	4.9%
	Post	263	11.16	2.2	−28.921 (262)	<0.001	⏺	

* Based on goal of 5 serves of vegetables per day (×5 weekdays). *Note:* Significant differences are highlighted with a black dot (i.e., ⏺).

**Table 10:** Increases, decreases and no change in fruit and vegetable consumption all schools combined.

		Fruit					Vegetables				
			Pre		Post			Pre		Post	
		*n*	Mean Week	Mean Daily	Mean Week	Mean Daily	*n*	Mean (Week)	Mean Daily	Mean (Week)	Mean Daily
All Schools	Increase	125	8.2	1.6	13.7	2.7 ^a^	130	7.7	1.5	14.6	2.9 ^c^
	No change	23	9.1	1.8	9.1	1.8	20	7.5	1.5	7.4	1.5
	Decrease	112	14.6	2.9	9.0	1.8 ^b^	100	15	3	8.1	1.6 ^d^

^a^ Significant increase (t(124) = 14.876, *p* < 0.001); ^b^ Significant decrease (t(124) = 11.931, *p* < 0.001); ^c^ Significant increase (t(124) = 16.201, *p* < 0.001); **^d^** Significant decrease (t(124) = 12.898, *p* < 0.001).
